# Community faecal carriage of extended-spectrum beta-lactamase-producing *Enterobacteriaceae* in french children

**DOI:** 10.1186/1471-2334-12-315

**Published:** 2012-11-21

**Authors:** André Birgy, Robert Cohen, Corinne Levy, Philippe Bidet, Céline Courroux, Mohamed Benani, Franck Thollot, Edouard Bingen

**Affiliations:** 1 Laboratoire associé au Centre National de Référence Escherichia coli et Shigelles, Service de Microbiologie, Hôpital Robert-Debré (AP-HP), Université Denis Diderot, Sorbonne, Paris Cité, Paris, F-75505, France; 2 ACTIV (Association Clinique et Thérapeutique Infantile du Val de Marne), St Maur Des Fossés, France and Centre Hospitalier Intercommunal de Créteil, 40 Avenue de Verdun, Créteil, 94000, France; 3 (Association Française de Pédiatrie Ambulatoire), Essey Les Nancy, France; 4 Service de Microbiologie, Hôpital Robert-Debré (AP-HP), 48 boulevard Serrurier CEDEX 19, Paris, 75935, France

**Keywords:** Community carriage, Extended-Spectrum Beta-Lactamase, *Enterobacteriaceae*, Children

## Abstract

**Background:**

The increasing incidence of community acquired infection due to Extended-Spectrum Beta-Lactamase (ESBL) -Producing *Enterobacteriaceae* represent a great concern because there are few therapeutic alternatives. The fecal flora of children in the community can represent a reservoir for ESBLs genes which are located on highly transmissible plasmids and the spread of these genes among bacterial pathogens is concerning. Because intestinal carriage is a key factor in the epidemiology of ESBL-producing *Enterobacteriaceae,* the study of the prevalence of these resistant bacteria and risk factors in young children is of particular interest.

**Methods:**

We assessed the prevalence and risk factors of community-acquired faecal carriage of extended-spectrum-β-lactamase (ESBL)-producing *Enterobacteriaceae* in children aged from 6 to 24 months, by means of rectal swabbing in community pediatric practices. Child’s lifestyle and risk factors for carriage of resistant bacteria were noted.

**Results:**

Among the 411 children enrolled, 4.6% carried ESBL-producing *Enterobacteriaceae*. CTX-M-1, CTX-M-15 and CTX-M-14 were the predominant ESBLs. The 18 *E. coli* isolates were genetically heterogeneous. Recent third-generation oral-cephalosporin exposure was associated with a higher risk of ESBL carriage (AOR=3.52, 95% CI[1.06-11.66], p=0.04).

**Conclusions:**

The carriage rate of ESBL-producing *Enterobacteriacae* in young children in the French community setting is noteworthy, underlining the importance of this population as a reservoir. Exposure to third-generation oral cephalosporins was associated with a significant risk of ESBL carriage in our study. Because of the significant public health implications including the treatment of community-acquired urinary tract infections, the spread of organisms producing ESBLs in the community merits close monitoring with enhanced efforts for surveillance.

## Background

In the past decade there has been an alarming upsurge in antibiotic-resistant *Enterobacteriaceae* producing extended-spectrum β-lactamases (ESBL), due at least partly to overuse of broad-spectrum cephalosporins [1]. The spread of mobile ESBL genes among bacterial pathogens is of great concern, not only because these enzymes confer resistance to oxy-imino-cephalosporins and other β-lactam antibiotics, but also because they are located on plasmids that confer resistance to other antimicrobial agents, leaving very few treatment options. CTXM–type ESBLs have now supplanted TEM and SHV-type ESBLs both in nosocomial and community settings [1] and *Escherichia coli* has replaced *Klebsiella spp.* as the predominant species of ESBL-producing *Enterobacteriaceae*[1].

Until recently, most infections caused by ESBL-producing *E. coli* were hospital-acquired. *bla*_*CTX-M*_ genes originate from environmental bacteria but have migrated to highly transmissible plasmids, which have been linked to ESBL circulation in the community. The community can thus represent a reservoir for ESBLs not yet detected in clinical isolates [2]. Colonisation in the intestinal compartment by ESBL-producing isolates has been associated with a high risk for developing infection due to ESBL producers [3]. Prevalence of ESBL-carriage in children varies depending on studies and geographical areas. It ranges from 0,1% in Bolivie and Perou [4] to 31% in Niger [5]. Because intestinal carriage is a key factor in the epidemiology of ESBL-producing *E. coli* infection [6], we investigated the prevalence of community-acquired faecal carriage of ESBL-producing *Enterobacteriaceae* in children aged between 6 and 24 months presenting to community paediatricians.

## Methods

This work was an ancillary study of a nasopharyngeal carriage study conducted in France following implementation of the 7-valent pneumococcal conjugate vaccine [7]. Between October 2010 and March 2011, 18 French pediatricians located in three regions (Ile de France, Lorraine, and Provence-Alpes-Côte d’Azur) took part in this prospective study.

A rectal sample was obtained from children aged 6 to 24 months, either during routine check-ups with normal findings, or when they presented with acute otitis media (AOM). The exclusion criteria were antibiotic treatment whatever the antibiotic was within 7 days before enrolment and severe underlying disease. Once their written informed consent had been granted, we queried the parents or guardians on the child’s demographics, risk factors for carriage of resistant bacteria including use of any antibiotics (between 7 days to 3 months before enrolment), daycare modalities (daycare center, home or child minder), previous hospitalization (during the previous 6 months), and immunization history (pneumococcal conjugate vaccine). The study was approved by the Saint Germain en Laye Hospital Ethics Committee.

On inclusion, rectal samples were taken with a flexible, sterile, soft rayon swab tip. After sampling, the swabs were immediately inoculated in transport medium (Copan, Brescia, Italy) at room temperature and sent within 48 hours to the National *E. coli and Shigella* Reference Center-associated laboratory at Robert Debré Hospital, Paris. The rectal swabs were spread on ChromID ESBL screening medium to screen the stool flora for cefpodoxime resistance (bioMérieux, La Balme-les-Grottes, France). ESBL detection was performed using the double-disk synergy test between clavulanic acid and extended spectrum-cephalosporins (ceftazidime and cefotaxime) [8]. Bacterial identification was performed with the API20E system (bioMérieux, Marcy l’Etoile, France).

Multiplex PCR was used to characterize ß-lactamase genes (including *bla*_*CTX-M*_*, bla*_*SHV*_*, bla*_*TEM*_*and bla*_*OXA-*1_), with previously described methods and primers. Amplicons were then sequenced [9,10]. Clonal relationships among *E. coli* isolates were identified by semi-automated rep-PCR (Diversilab, bioMérieux, France) [11]. The strains were assigned to one of the four main *E. coli* phylogenetic groups (A, B1, B2 and D), using a previously described multiplex PCR-based method [12].

Data were double-entered using 4D software (version 6.4), and analyzed using Stata SE 9.1 (Stata Corp., College Station, TX, USA) for univariate analysis and multivariate logistic regression (odds ratios [ORs] and 95% confidence intervals [CI]). The Pearson Chi-square test was used in univariate analysis to identify factors related (p<0.10) to ESBL-producing *Enterobacteriaceae* carriage. Variables identified by univariate analysis were age (continuous variable after testing linearity), the study group (healthy controls vs children with AOM) and recent antibiotic treatment (within 3 months before enrolment).

## Results

ESBL carriage was assessed in 411 children none of whom were born preterm. Mean age of the children was 13.3±6.1 months, the sex ratio was 1.07 (M/F). 37.2% of the children attended daycare centers, others were cared for at home, 54.5% had siblings, and 34.1% had received antibiotics within 7 days to 3 months before enrolment. Third-generation oral cephalosporins (O3GC) represented 25% of the antibiotics prescribed and especially cefpodoxime proxetil. ESBL-producing *Enterobacteriaceae* were found in 19 patients (4.6%): 1 patient with *E. coli* and *Citrobacter freundii*, 1 with *K. pneumoniae*, and 17 with *E. coli.* None of these children had been hospitalized in the previous 6 months. ESBL-producing *Enterobacteriaceae* carriage was not related to daycare modalities. The rate of ESBL-producing *Enterobacteriaceae* carriage was higher in children with recent O3GC exposure (11.1%) than in those without recent antibiotic exposure (4.4%) or with recent aminopenicillin exposure (3.3%) (univariate analysis: OR=3.00, 95% CI [0.94–9.58]). The risk of ESBL-producing *Enterobacteriaceae* carriage was also higher among children over 1 year old than in younger children (6.5% versus 2.5%, respectively; OR=2.69, 95% CI [0.95–7.61]).

**Figure 1:**
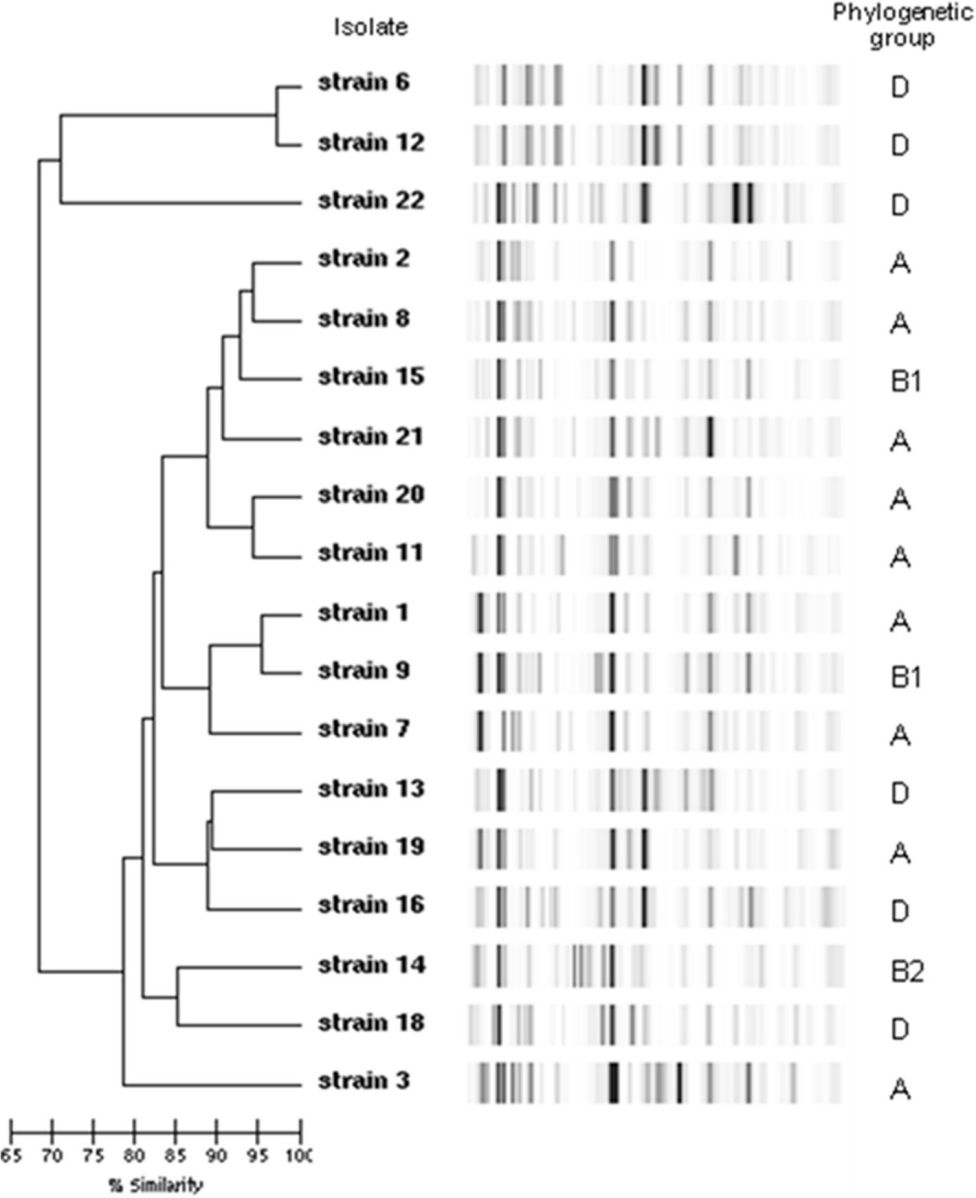
**Genetic diversity of ESBL-producing *E. coli*.** Dendrogram showing the genetic heterogeneity of ESBL-producing *E. coli*. 17 different profiles were found among the 18 isolates with a cut-off for genetic relation of 97%.

After adjustment in a logistic regression model, recent O3GC use was associated with a significant risk of ESBL carriage (Adjusted Odds Ratio (AOR) =3.52, 95% CI [1.06-11.66], p=0.04). Table 1 shows the bacterial species, the *E. coli* phylogenetic groups, the ESBLs produced, other ß-lactamase genes, and associated resistance patterns. Phylogenetic group A (9/18) predominated, and the most frequent ESBL was CTX-M-1 (9/20). 11/16 of the CTX-M-producing *E. coli* carried one other β-lactamase-encoding gene (mainly TEM-1) and 14/16 were resistant to at least one other antibiotic family. Figure 1 shows the genetic heterogeneity of the ESBL-producing *E. coli*; 17 different profiles were found among the 18 isolates with a cut-off for genetic relation of 97%.

**Table 1:** **Bacterial species, phylogenetic group of *E. coli*, ESBL produced, others ß-lactamase genes and associated resistance**

**Isolate number**	**Strains**	**Phylogenetic group**	**ESBL type**	**Other beta-lactamase type**	Associated resistance patterns*
1	*E. coli*	A	CTX-M-1	TEM-135	NONE
2	*E. coli*	A	CTX-M-1		TSU,NA
7	*E. coli*	A	CTX-M-1	TEM-1	TSU, GT
19	*E. coli*	A	CTX-M-1	TEM-1	TSU, NA, CIP
20	*E. coli*	A	CTX-M-1	TEM-1	TSU, NA, KA
8	*E. coli*	A	CTX-M-15	TEM-1	TSU, NA, GT,TM
11	*E. coli*	A	CTX-M-15	OXA-1	TSU, NA, CIP, K, GT, TM
3	*E. coli*	A	SHV-12		NA
21	*E. coli*	A	TEM-52		TSU, NA
9	*E. coli*	B1	CTX-M-1	TEM-1	TSU
15	*E. coli*	B1	CTX-M-1		TSU
14	*E. coli*	B2	CTX-M-15		TSU, NA, CIP, K, GT, TM
13	*E. coli*	D	CTX-M-1		TSU, NA, CIP
22	*E. coli*	D	CTX-M-1	TEM-1	TSU, NA
6	*E. coli*	D	CTX-M-14		NA
12	*E. coli*	D	CTX-M-14	TEM-1	NA
18	*E. coli*	D	CTX-M-27	TEM-1	TSU, NA, CIP
16	E. coli**	D	CTX-M-32	TEM-1	NONE
	C. freundii**		CTX-M-32		NONE
	*K. pneumoniae*		CTX-M-14	SHV-1	TSU

*TSU, trimethoprim-sulfamethoxazole; *NA*, nalidixic acid; *CIP*, ciprofloxacin; *GT*,gentamicin; *KA*, kanamycin; *TM*, tobramycin.

**same patient.

## Discussion

ESBL-producing *Enterobacteriaceae* are recognized as important nosocomial pathogens in children, and are often associated with outbreaks [13]. Screening for intestinal carriage is crucial to predict the risk of ESBL infection, as the colon serves as a reservoir for extra-intestinal pathogenic *E. coli*[14-16]*.* ESBL-producing *E. coli* have previously been described as community-acquired in adult patients [17]. The rate of ESBL-producing- *E. coli* carriage in healthy subjects were 3.7% and 5.8% in a Spanish [2] and Swiss study [18], respectively. Detection of ESBL in fecal isolates from healthy children has also been previously reported. Pallecchi et al. detected ESBL-producing *Enterobacteriaceae* in 4 (0.1%) of 3208 children, and molecular characterization revealed the presence of CTX-M type β-lactamase genes in the isolates from all 4 children [4]. Recently, Guimaraes et al. studied fecal carriage of ESBL-producing *Enterobacteriaceae* in 112 healthy children and found that 3 (2.6%) harbored ESBL-producing *E. coli* (CTX-M-1, TEM-52 and SHV-12) [19]. These isolates belonged to phylogenetic groups A, B1 and D, respectively [19]. In our study, 4.6% (19/411) of children aged from 6 to 24 months carried ESBL-producing *Enterobacteriaceae*. The prevalence of carriage of ESBL-producing *Enterobacteriaceae* is probably underestimated in this study because children with antibiotic treatment within 7 days before enrolment and severe underlying disease were excluded from the study. However, the percentage of children carrying ESBL-producing *Enterobacteriaceae* strains in our population of children is comparable to the percentage of ESBL-producing-*E. coli* isolated from urinary tract infection in pediatric patients found in France (data on file). Most of the isolates of our study (18/20, 90%) produced a CTX-M type ESBL, and were resistant to multiple antibiotic classes (Table 1). Virulent extra-intestinal strains of *E. coli* belong to phylogenetic group B2 and, to a lesser extent, group D, whereas commensal strains mainly belong to group A or B1 [20]. Most of our ESBL-producing *E. coli* isolates belonged to group A/B1, possibly because of greater antibiotic exposure of group A/B1 strains belonging to the fecal flora [21]. Interestingly, one group A *E. coli* strain isolated here carried a TEM-52 ESBL. Neonatal meningitis caused by TEM-52-producing group A *E. coli* was recently described [13].

Rep-PCR showed a high level of genomic diversity among the 18 *E. coli* isolates, illustrating the high capacity for spread among different genetic backgrounds found in the community, via mobile conjugative elements. Exposure to third-generation oral cephalosporins was associated with a significant risk of ESBL carriage in our study (AOR=3.52, 95% CI [1.06-11.66], p=0.04). These results support efforts to reduce prescribing of cephalosporins for control of resistant *Enterobacteriaceae* not only for the treatment of urinary tract infection (UTIs) but also upper respiratory infection.

## Conclusion

The carriage rate of ESBL-producing *Enterobacteriaceae* in young children in the French community setting (4.6%) is noteworthy, underlining the importance of this population as a reservoir [22].

Exposure to third-generation oral cephalosporins was associated with a significant risk of ESBL carriage in our study. Because of the significant public health implications including the treatment of community-acquired UTIs, the spread of organisms producing ESBLs in the community merits close monitoring with enhanced efforts for surveillance.

## Competing interests

All authors: no conflicts.

## Authors' contribution

AB: carried out the molecular genetic studies of bacterial strains and drafted a part of the manuscript. RC: have been involved in designing the study, collecting samples and informations, analysing data, revising critically the article and drafting a part of the manuscript. Have given final approval of the version to be published. CL: have been involved in designing the study, collecting samples and informations, analysing data, revising critically the article and drafting a part of the manuscript. Have given final approval of the version to be published. PB: have been involved in the molecular genetic studies, in the sequence alignement and in interpretation of data. CC: participated in the molecular genetic studies and in collection of samples and data. MB: was one of the main investigators of the study and have been involved in designing the study. FT: was one of the main investigators of the study and have been involved in designing the study. EB: have been involved in designing the study, analysing data, drafting a part of the manuscript and revising critically the article. Have given final approval of the version to be published. All authors read and approved the final manuscript.

## Authors' information

EB is a French microbiologist specialist, scientific director of a research unit (EA3105) and head of the microbiologic department of Hopital Robert-Debré, pediatric hospital. He is also director of the associated laboratory of the national center of reference of shiga toxin-producing *E. coli*. His main research interests are epidemiologic studies and clinical trials in community and hospital acquired infections including those due to Extended-Spectrum-beta-lactamases-producing *Enterobacteriaceae*. He has published more than 250 papers in English language in these fields.

## Pre-publication history

The pre-publication history for this paper can be accessed here:

http://www.biomedcentral.com/1471-2334/12/315/prepub
